# Temporal and spatial dynamics of *Fusarium* spp. and mycotoxins in Swedish cereals during 16 years

**DOI:** 10.1007/s12550-022-00469-9

**Published:** 2022-10-24

**Authors:** Ida Karlsson, Eva Mellqvist, Paula Persson

**Affiliations:** 1 Department of Crop Production Ecology, Swedish University of Agricultural Sciences, Box 7043, 750 07 Uppsala, Sweden; 2 Swedish Board of Agriculture, Klostergatan 13, 532 30 Skara, Sweden; 3 Present Address: Clinical Genomics Uppsala, Dept. of Immunology, Genetics and Pathology, Uppsala University, Rudbeck Laboratory, 751 85 Uppsala, Sweden

**Keywords:** *Fusarium*, Mycotoxins, Deoxynivalenol, Zearalenone, Nivalenol, HT-2 and T-2

## Abstract

We analysed the dynamics of *Fusarium* spp. and mycotoxin contamination in Swedish cereals during 2004–2018. More than 1400 cereal samples from field trials were included, collected in a monitoring programme run by the Swedish Board of Agriculture. Five *Fusarium* mycotoxins were quantified with LC-MS/MS and fungal DNA from four species was quantified using quantitative real-time PCR. Correlation analyses revealed that deoxynivalenol (DON) and zearalenone (ZEN) were mainly associated with *Fusarium graminearum*, but stronger correlations with *F*. *culmorum* was seen some years. Nivalenol (NIV) was associated with *F*. *poae* and the HT-2 and T-2 toxins with *F. langsethiae*. Clear differences in mycotoxin contamination between different cereal crops and geographical regions were identified. The highest levels of DON and ZEN were found in spring wheat in Western Sweden. For NIV, HT-2 and T-2 toxins, the levels were highest in spring oats and spring barley. Regional differences were not detected for NIV, while HT-2 and T-2 toxins were associated with the northernmost region. We found that delayed harvest was strongly associated with increased levels of DON and ZEN in several crops. However, harvest date did not influence the levels of NIV or HT-2 and T-2 toxins. Our results suggest similar distribution patterns of DON and ZEN, in contrast to NIV and HT-2 and T-2 toxins, probably mirroring the differences in the ecology of the toxin-producing *Fusarium* species. Timely harvest is important to reduce the risk of DON and ZEN contamination, especially for fields with other risk factors.

**Supplementary Information:**

The online version contains supplementary material available at 10.1007/s12550-022-00469-9.

## Introduction

Plant pathogenic species within the fungal genus *Fusarium* cause the globally important *Fusarium* head blight disease in small-grain cereals. Some *Fusarium* pathogens or species associated with cereals may also cause severe mycotoxin contamination. The infection and mycotoxin production start already in the field, which may result in severe contamination at harvest.

*Fusarium* fungi produce several types of mycotoxins. In small-grain cereals in Northern Europe, *Fusarium* toxins deoxynivalenol (DON), nivalenol (NIV), zearalenone (ZEN), HT-2 and T-2 toxins have been of greatest concern (Hofgaard et al. 2016; Hietaniemi et al. 2016; Edwards 2017; Edwards and Jennings 2018). One *Fusarium* species may produce several toxins, and the same toxin may be produced by several species. DON and NIV are produced by different chemotypes of *F. graminearum* and *F. culmorum* (Pasquali et al. 2016) and these species may also produce ZEN (Munkvold 2017). Nivalenol may also be produced by *F. poae* (Pettersson 1991; Thrane et al. 2004) which is the main producer in the Nordic countries (Pettersson et al. 1995; Langseth and Rundberget 1999; Lindblad et al. 2013; Fredlund et al. 2013; Hietaniemi et al. 2016). *F. langsethiae* and *F. sporotrichioides* are known producers of the HT-2 and T-2 toxins (Torp and Langseth 1999; Thrane et al. 2004). However, *F. langsethiae* is the main producer of HT-2 and T-2 toxins in Northern Europe (Kosiak et al. 2003; Edwards et al. 2012; Fredlund et al. 2013; Hofgaard et al. 2016).

To protect consumer health, legislative limits for the above-mentioned mycotoxins have been introduced in many countries. In the European Union, maximum levels for DON and ZEN were set for cereals intended for human consumption. The maximum level of DON in unprocessed cereals is 1250 μg/kg, except for oats, maize and durum wheat where the maximum level is 1750 μg/kg (EC - European Commission 2006a). For ZEN, the maximum level is 100 μg/kg for unprocessed cereals (EC - European Commission 2006a) and 350 μg/kg for unprocessed maize (EC - European Commission 2007), respectively. For the sum of HT-2 and T-2 toxins, the European Commission has set guidance levels for unprocessed cereals of 1000 μg/kg for oats, 200 μg/kg for barley and 100 μg/kg for wheat, rye and other cereals (EC - European Commission 2013). Furthermore, guidance levels were set for DON, ZEN and the sum of HT-2 and T-2 toxins for unprocessed cereals intended to be used for feed (EC - European Commission 2006b). For NIV, no regulatory limits were set so far in EU, as it is considered to co-occur in low concentrations with DON. Thereby a specific measure for NIV is not regarded to be necessary to prevent humans from unacceptable exposures (EC - European Commission 2006a).

Mycotoxin contamination is strongly dependent on weather conditions, but agronomic factors are also of importance. Risk factors vary with mycotoxin, reflecting different biological and ecological characteristics of different toxin-producing *Fusarium* species. For example, for *F. graminearum* and *F. culmorum*, humid and warm weather during flowering when the crop is the most susceptible increases the risk of infection, as well as no till and maize as a preceding crop (Lacey et al. 1999; Dill-Macky and Jones 2000; Beyer et al. 2006). In contrast, risk factors for contamination with *F. langsethiae* are less well-known, but there are indications that it is favoured by dryer and warmer conditions (Imathiu et al. 2013). Recommendations to reduce mycotoxin contamination in small grain cereals include crop rotation with non-susceptible crops, ploughing, selection of more resistant varieties and timely fungicide treatment (EC - European Commission 2006c; Ferrigo et al. 2016). It is also recommended to avoid delayed harvest which has been linked to higher levels of DON and ZEN (Edwards and Jennings 2018). However, there is little knowledge on how harvest date affects other *Fusarium* toxins.

Surveys in Europe have shown significant regional and yearly fluctuations in disease occurrence and mycotoxin contamination (for example: Landschoot et al. 2012; Birr et al. 2020; Pallez-Barthel et al. 2021; Van der Fels-Klerx et al. 2021). This variation has been explained by varying climatic conditions or agricultural practices, and different geographical distribution of *Fusarium* species and isolates. For instance, the 15-ADON chemotype of *F. graminearum* is more widespread in Southern and Western Europe while the 3-ADON chemotype dominates in the Northern and Eastern part of Europe (Pasquali et al. 2016).

In Sweden, high loads of *Fusarium* mycotoxin contamination has occurred some years during the last decade, causing high economic loss and problems for farmers. The Swedish Board of Agriculture has run a monitoring programme since 2004, taking yearly samples subjected to mycotoxin analyses and quantification of *Fusarium* DNA. More than 1400 samples from five cereal species have been collected until 2018. The aim of the study was to get knowledge about the temporal and spatial patterns of *Fusarium* toxin contamination in relation to *Fusarium* spp. infection in different cereal crops produced in the main agricultural areas of Sweden. A better insight in the consequences of cereal infections with *Fusarium* spp. on the quality of harvest products under Nordic climatic conditions may improve risk assessment to limit *Fusarium* toxins entering the human food chain*.* For instance, such knowledge can be used to develop or validate forecasting models that can assist farmers in selecting appropriate preventative measures.

## Material and methods

### Sample and data collection

Samples were collected in a monitoring programme targeting *Fusarium* fungi and mycotoxins carried out by the Swedish Board of Agriculture. Samples were collected from official field trials managed by the Rural Economy and Agricultural Societies and the Swedish University of Agricultural Sciences (SLU). Trials included variety trials (*n* = 759), plant protection trials (*n* = 322), soil tillage/crop rotation trials (*n* = 47) and plant nutrient trials (*n* = 17). For 270 samples, the trial type was not recorded. Trials had a block design and were located in farmers’ fields or in experimental farms. The sampling was designed by the Swedish Board of Agriculture to represent different cereal crops, regions and the most common varieties grown by farmers. Samples were taken from untreated plots or fungicide-treated plots where the treatment was not directed towards *Fusarium*. In total, 1415 samples of winter wheat, spring wheat, spring barley, winter triticale and spring oats were collected during 2004–2018 (Table 1).

**Table 1:** Overview of samples collected and climate and soil characteristics of five regions in Sweden 2004–2018

**Number of samples**							**Mean**				
	Spring barley	Spring oats	Winter triticale	Spring wheat	Winter wheat	Sum	Annual temperature (°C)	Annual precipitation (mm)	Clay content (%)	Sand content (%)	Silt content (%)
**Southwest**	60	32	37	47	298	474	7.8	685	18	48	34
**West**	48	54	37	30	148	317	6.3	711	27	27	46
**Southeast**	11	15	20	13	37	96	7.3	531	16	47	37
**East**	40	32	31	57	173	333	6.3	587	34	22	44
**North**	18	31	19	42	85	195	5.9	570	41	12	47
Sum	177	164	144	189	741	1415					

**Fig. 1:**
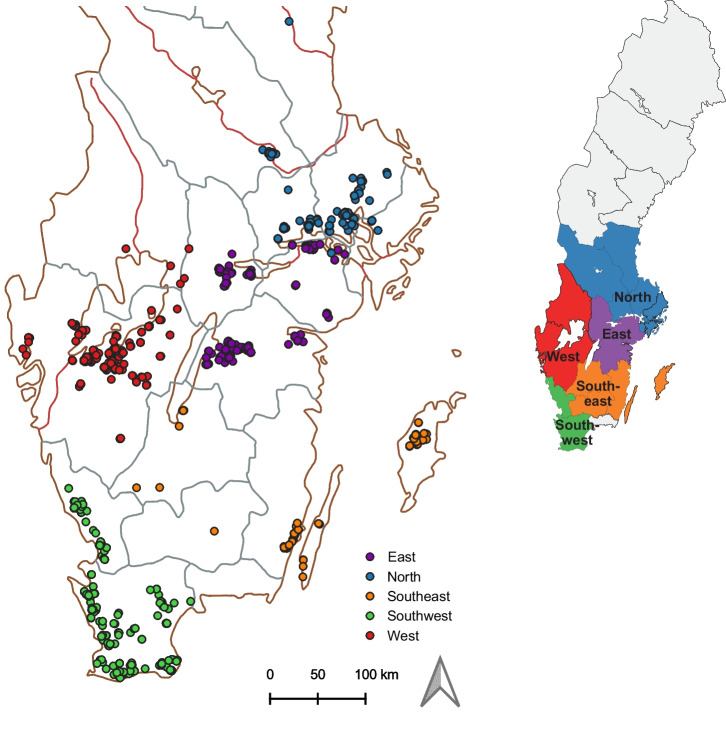
Map of sampled field trials in five regions in Sweden. The map includes 1173 of 1415 trials for which coordinates were available

Samples were taken from trials in 20 counties which were grouped into five larger regions representing main agricultural areas in Sweden: ‘Southwest’, ‘Southeast’, ‘West’, ‘East’ and ‘North’ (Fig. 1). GPS coordinates for each trial were retrieved from the SLU Fältforsk database (https://www.slu.se/faltforsk) and were available from 1173 trials (Fig. 1). Annual precipitation and annual temperature for each coordinate were extracted from the Bioclim raster dataset (version 1.4) obtained from the Worldclim database (https://www.worldclim.org/). These data were used to calculate mean annual precipitation and mean annual temperature for 2004–2018 for each region. Similarly, clay, sand and silt contents for each coordinate were extracted from the digital soil map of Sweden (Piikki and Söderström 2019). Subsequently, the mean clay, sand and silt content for each region was calculated.

Mature cereal grain was collected with the trial combine harvesters where a subsample of half a litre per plot was collected continuously during harvesting. Samples were dried to 15% water content within 24 h. Around 100 g grain was taken from each harvested sample for the mycotoxin and DNA analyses. Dried grain samples were stored at room temperature for up to 2 months before further analysis.

### Mycotoxin analyses

The amount of five *Fusarium* mycotoxins was quantified yearly for all samples at harvest. Quantification of DON, NIV, ZEN, HT-2 and T-2 was carried out using liquid chromatography–double mass spectrometry (LC–MS/MS) at Århus University, Flakkebjerg, Denmark (Etzerodt et al. 2016).

#### Chemicals

Mycotoxin standards (unlabelled and 13C22-labelled) were purchased from Biopore. MS-grade solvents acetonitrile and methanol were supplied by Fisher Scientific (Loughborough, UK). MilliQ water (resistivity 18.2 Mohm × cm, TOC less than 1 µg/l) was produced in-house with a Milli-Q® Advantage A10 Water Purification System from Merck KGaA (Darmstadt, Germany).

#### Extraction

Dry seeds were homogenized in a Waring blender. Five-gram homogenized sample was weighed in 50 ml Falcon tubes. Twenty-millilitre 80% acetonitrile was added and the mixture was ultrasonicated for 20 min, shaken by hand and stored in room temperature until next days. Then the mixture was shaken for 2 h in a shaking table, centrifuged and the supernatant was collected and stored at −20 °C until analysis in LC-MS/MS. Groups of 20 samples were created. In each group, three blank samples (no mycotoxins) were used for spiking experiments at 0 µg/kg, low (10 µg/kg) and high (50 µg/kg) concentrations for 4 mycotoxins and 0 µg/kg, 2 µg/kg and high (10 µg/kg) for zearalenone. The results of the spiking experiments were later used for determining recovery percentage.

#### LC-MS/MS analysis

A hundred microlitres of the supernatant was supplemented with 350 µl MilliQ water and 50 ml of the internal standard mix (25 µg/l for 4 mycotoxins and 5 µg/l for zearalenone). Seed sample extracts were analysed on an Agilent 1100 HPLC system coupled with a 3200 QTRAP mass spectrometer (AB SCIEX, Foster City, California) both in negative and positive modes. The separations were performed on a 250 mm × 2.1 mm id 5 μm BDS Hypersil C18 column (Fisher Scientific). The samples were injected (10 µl) onto a pre-heated column at 26 °C, with a flow rate of 200 μl/min.

Samples were analysed for mycotoxin contents using the mobile phases of eluent A (1% methanol in water) and eluent B (100% acetonitrile in water) in both positive and negative ionization modes. The chromatographic gradient in positive mode was 0–0.5 min 50% A, 0.5–8 min decreasing to 0% A, 8–11 min kept at 0% A, 11–12 min back to 50% A, 12–17 equilibrating and 50%. The chromatographic gradient in negative mode was 0–1 min 100% A, 1–4 min decreasing to 0% A, 4–14 min kept at 0% A, 14–14.5 min back to 100% A, 14.5–23 min equilibrating and 100%.

Analyst Software (AB Sciex Foster City, California) was used for instrument control, data acquisition and subsequent quantifications. Mass spectrometry parameters are found in Online Resource 1. For quantification, calibration curves were obtained using 5 standard solutions in the range of 1–100 ng/ml (except zearalenone for which a range of 0.2–20 ng/ml was used). The concentration in the samples was calculated on basis of the standard curve taking into consideration the signals from the internal standards.

The above-described method was used since 2009. In the period 2004–2009, a very similar chromatographic method was used on a Hewlett-Packard 1100 system. MS/MS detection was performed with an Applied Biosystems Sciex API 2000 (Nielsen et al. 2011). The extraction method used in 2004–2009 included an SPE purification/concentration step that was needed because the 2000 instrument had lower sensitivity than the later used 3200 instrument.

The limits of quantification (LOQs) for the mycotoxins were 10 μg/kg for DON and NIV; 2–5 μg/kg for ZEN; and 5–10 μg/kg for HT-2 and T-2 toxins.

### *Fusarium* spp. quantification

Each year, a subset of samples were also subjected to real-time qPCR to quantify the amount of different *Fusarium* species. Samples with higher mycotoxin content representing different crops and regions were selected. The species surveyed varied over the years, from 2005 to 2009, three species were analysed: *F*. *graminearum*, *F*. *culmorum* and *F*. *poae*. DNA extractions and real-time qPCR using TaqMan assays were carried out by the Swedish Food Agency, Uppsala, Sweden, during 2006 (Fredlund et al. 2008), and by Plant Research International, Wageningen, the Netherlands, the other years (Waalwijk et al. 2004).

From 2010 to 2018, three species were targeted: *F*. *graminearum*, *F*. *culmorum* and *F*. *langsethiae*. DNA extractions and real-time qPCR were carried out at Århus University, Flakkebjerg, Denmark. From the milled sample used for mycotoxin analysis, a subsample of 5 g was taken. The subsample was grinded again using liquid nitrogen and a GenoGrinder (SPEX SamplePrep, USA) using 8 steel balls (ø 4–5 mm) for 3 × 30 s at 1300 rpm. DNA was extracted from 25 mg of powdered grain suspended in 350 µl lysis buffer using a mag maxi kit (LGC Genomics, GmbH, Germany) following the manufacturer’s instructions, automated with a KingFisher™ Magnetic Particle Processor (Thermo Fisher Scientific, USA). Extraction was performed on 3 × 130 µl lysate, and DNA was eluted in 100 µl elution buffer. Before qPCR, the extracted DNA was diluted 1:10 and qPCR was performed with SYBR Green assays as described previously (Nicolaisen et al. 2009). Fungal DNA was quantified relatively to plant DNA in order to account for variation in moisture and DNA extraction efficiency. Results were expressed as pg fungal DNA/µg plant DNA.

### Statistical analyses

Linear mixed models were used to test differences in mycotoxin contamination between different crops and regions. Year and the interactions between year, crop and region were included as random factors. When several samples were taken from the same trial (e.g., different varieties), the mean mycotoxin content per trial was calculated. Linear mixed models were fitted for each toxin using the *lmer* function in the ‘lme4’ package (Bates et al. 2015). Significance was assessed using type III ANOVA with Wald *F*-tests using the *Anova* function in the ‘car’ package (Fox and Weisberg 2019). Pairwise comparisons were made with the *emmeans* function in the ‘emmeans’ package 1.4.6 using Tukey’s test adjusted for multiple testing.

The effect of harvest week (calendar week) on mycotoxin contamination was analysed with linear mixed models as described above. Region, year and their interaction were included as random variables in these models. The different crops were analysed separately.

The relationship between the different mycotoxins and *Fusarium* species was analysed using Pearson’s correlation analysis.

Samples with mycotoxin levels under the LOQs were assigned a value of LOQ/2. Both mycotoxin and *Fusarium* data were transformed with the natural logarithm (ln) to approach homoscedasticity and a normal distribution of the data. For *Fusarium* DNA concentrations, a value of 1 was added to each observation before ln transformation.

All statistical analyses were carried out in R 4.1.1 (R Core Team 2014).

## Results

### Level of mycotoxin contamination and yearly variations

Mycotoxins were frequently detected in the 1415 samples of harvested cereal grains collected between 2004 and 2018 from field trials in Sweden. DON was the most frequent toxin and was detected in ~1000 samples, followed by NIV, ZEN and HT-2 and T-2 toxins (Table 2).

**Table 2:** Occurrence of *Fusarium* mycotoxins in five cereal species in Sweden 2004–2018

	**% > LOQ**	**Median**	**Mean**	**Max**	**95th percentile**
**DON**					
Spring barley	91	75	162	1980	566
Spring oats	88	126	445	10596	1435
Winter triticale	93	144	666	11990	2717
Spring wheat	76	68	446	18250	1034
Winter wheat	68	32	126	4441	476
**ZEN**					
Spring barley	25	2.5	17	887	47
Spring oats	14	2.5	73	7384	45
Winter triticale	37	2.5	15	434	60
Spring wheat	17	2.5	5	54	29
Winter wheat	25	2.2	9	485	33
**NIV**					
Spring barley	66	17	44	927	132
Spring oats	71	21	65	1000	234
Winter triticale	24	5	9	61	27
Spring wheat	35	5	15	265	52
Winter wheat	43	5	18	696	67
**HT-2 + T-2**					
Spring barley	46	10	21	340	78
Spring oats	59	19	66	1165	208
Winter triticale	8	5	9	314	14
Spring wheat	3	5	7	77	8
Winter wheat	8	8	8	79	14

^*^Highest LOQs during the period were 10 µg/kg for deoxynivalenol (DON), 5 µg/kg for zearalenone (ZEN), 10 µg/kg for nivalenol (NIV) and 20 µg/kg for the sum of HT-2 and T-2 toxins. For calculations, samples < LOQ were assigned LOQ/2

**Fig. 2:**
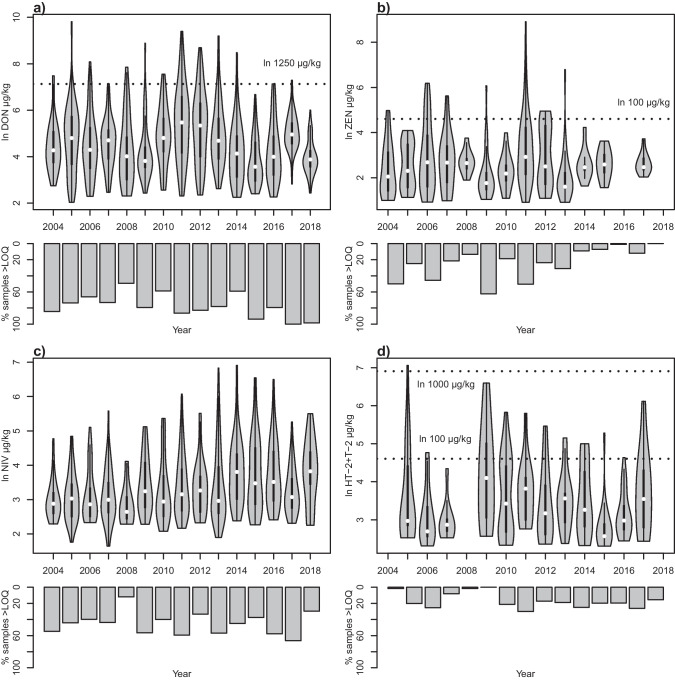
Yearly variation of mycotoxin content in Swedish cereals 2004–2018. **a** Deoxynivalenol (DON), **b** zearalenone (ZEN), **c** nivalenol (NIV) and **d** the sum of HT-2 and T-2 toxins. For each toxin, the upper panel shows mirrored density curves of mycotoxin levels above the highest level of quantification (LOQ) used during the period. The width of the density curves corresponds to the frequency of data points. Highest LOQs were 10 µg/kg for DON, 5 µg/kg for ZEN, 10 µg/kg for NIV and 20 µg/kg for the sum of HT-2 and T-2 toxins. White dots indicate medians and the thicker black bars indicate the interquartile ranges. The lower panels show the percentage of samples below LOQ. Dotted lines indicate current legislative limits for DON and ZEN and guidance levels for HT-2 and T-2 toxins. Mycotoxin data are transformed with the natural logarithm (ln)

Few samples exceeded the legislative limits (or for HT-2 and T-2 toxins, the guidance levels). For DON, 43 (3.4%) of the non-oats samples out of 1415 exceeded the legislated upper limit of 1250 µg/kg, and 7 (4.3%) spring oat samples exceeded the limit of 1750 µg/kg for oats (Fig. 2). For ZEN and the sum of HT-2 and T-2 toxins, the numbers exceeding their respective limits of 100 µg/kg cereal were 31 (2.2%) and 40 (2.8%), respectively (Fig. 2). Only one sample (spring oats) exceeded 1000 µg/kg of the sum of HT-2 and T-2 toxins.

**Fig. 3:**
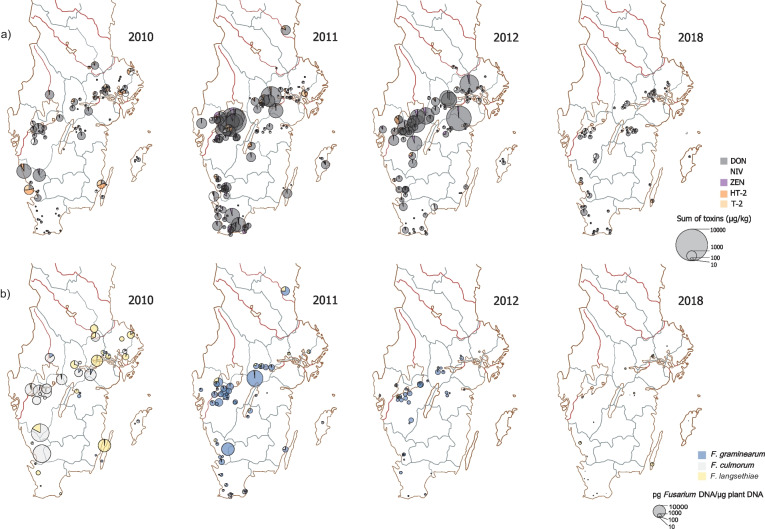
Spatial distribution of **a** mycotoxin and **b** *Fusarium* spp. contamination of Swedish cereal samples for selected years with contrasting levels of DON contamination. The area of the pie charts is proportional to the sum of mycotoxins or *Fusarium* DNA content in the sample respectively. DON, deoxynivalenol; ZEN, zearalenone; NIV, nivalenol

Both yearly and geographical variation for the four mycotoxins was apparent. As an example of the geographical variation, the mycotoxin compositions in cereal samples in some years with contrasting level of DON contamination are illustrated in Fig. 3a. Median DON levels varied significantly over the years with for example low medians 2008 and 2018, and higher levels 2011 and 2012 (Figs. 2a and 3a). For ZEN, a higher median was observed in 2011 for example, similar to DON, but very few samples exceeded LOQ in 2016 and 2018 (Fig. 2b). NIV was low in 2008 and higher for instance in 2014 and 2018 — 2 years with dry and warm summers (Fig. 2c). For the sum of HT-2 and T-2, the highest median was in 2009 (Fig. 2d). Thus, the toxins vary in importance, with the different toxins reaching higher levels different years.

### Mycotoxin contamination in different crops and regions

**Fig. 4:**
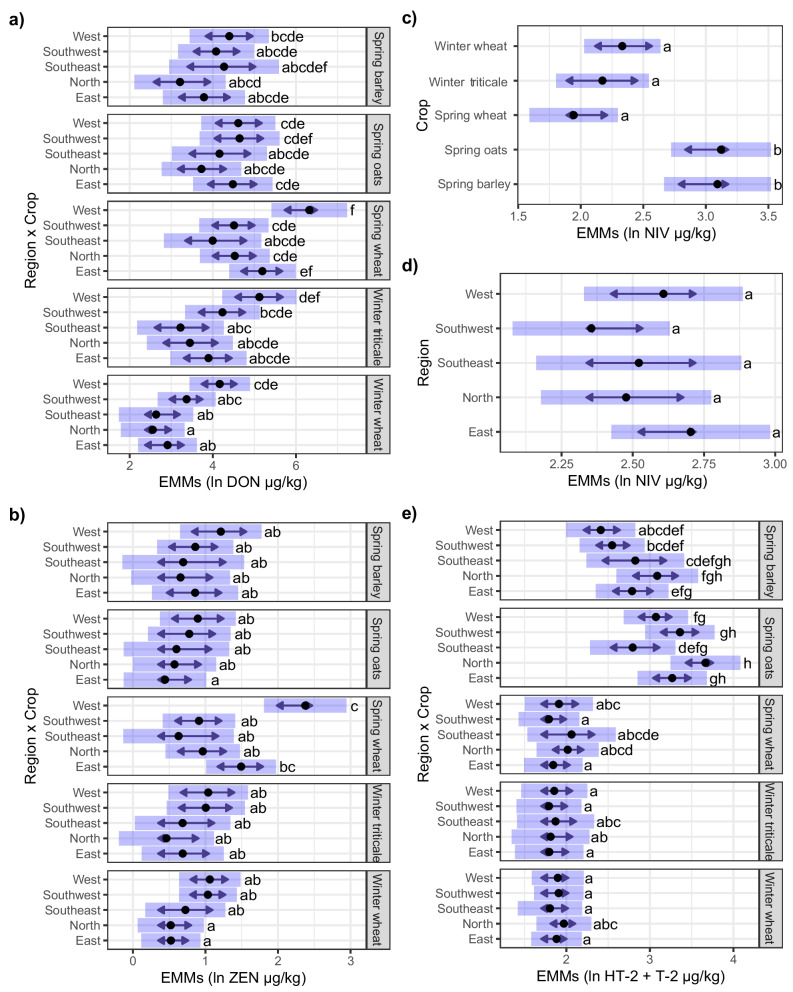
Estimated marginal means (EMMs) of mycotoxin content depending on crop and region for **a** deoxynivalenol (DON), **b** zearalenone (ZEN), **c** and **d** nivalenol (NIV) and **e** the sum of HT-2 and T-2 toxins. EMMs are indicated by black dots, bars indicate 95% confidence intervals and arrows are for comparing groups. Non-overlapping arrows indicate significant differences (*p* < 0.05) adjusted for multiple testing. Mycotoxin data are transformed with the natural logarithm (ln)

There were significant differences in mycotoxin levels between different cereal crops and regions (Fig. 4 and Table 3). For DON, ZEN, HT-2 and T-2 toxins, there was a significant interaction between region and cereal crop, meaning that the pattern of toxin contamination in different crops was not the same in all regions. The highest levels of both DON and ZEN were found in spring wheat in region ‘West’, while the lowest levels of DON were found in winter wheat in the region ‘North’. Region ‘West’ also had elevated levels of DON in the two winter crops, triticale and winter wheat.

**Table 3:** Effect of region and crop on *Fusarium* mycotoxin contamination with deoxynivalenol (DON), zearalenone (ZEN), nivalenol (NIV) and HT-2 and T-2 toxins of Swedish cereals. Analysis of deviance table

		***F* value**	**Df**	**Res. Df**	***p* value**	
DON	Intercept	661.4	1	14	0.000	***
	Region	9.1	4	57	0.000	***
	Crop	14.4	4	47	0.000	***
	Region*Crop	2.0	16	173	0.016	*
ZEN	Intercept	80.9	1	15	0.000	***
	Region	5.2	4	60	0.001	**
	Crop	5.8	4	51	0.001	***
	Region*Crop	3.1	16	182	0.000	***
NIV	Intercept	1412.7	1	14	0.000	***
	Region	2.1	4	60	0.094	
	Crop	14.2	4	50	0.000	***
	Region*Crop	1.0	16	190	0.439	
HT-2 + T-2	Intercept	766.4	1	15	0.000	***
	Region	3.5	4	66	0.012	*
	Crop	47.1	4	48	0.000	***
	Region*Crop	2.0	16	210	0.016	*

^*^p < 0.05; **p < 0.01; ***p < 0.001

For NIV, the levels were the highest in spring oats and spring barley but there were no significant regional differences (Fig. 4d). For HT-2 and T-2 toxins, the levels were the highest in spring oats followed by spring barley. Region ‘North’ had higher levels than the other regions for both crops (Fig. 4e). The lowest levels of HT-2 and T-2 toxins were observed in region ‘West’ for spring barley and region ‘Southeast’ for spring oats. Regarding winter wheat, spring wheat and winter triticale, only minor differences in HT-2 and T-2 toxin levels were observed between the different regions.

There were differences in climate and soil characteristics in the different regions. In general, mean annual temperature as well as soil sand content decreased with latitude, while mean annual precipitation was greater in the western regions studied (Table 1).

### Effect of harvest week

The effect of harvest week on mycotoxin content was analysed for each crop and toxin (Table 4). Harvesting later increased the levels of DON in winter wheat, winter triticale and spring wheat (Table 4; Online Resource 2). For ZEN, a later harvest week was associated with increased toxin levels in winter wheat, winter triticale, spring wheat and spring oats. For DON, the model estimated a weekly increase in toxin levels of 20% in winter wheat, 48% in spring wheat and 62% in winter triticale. For ZEN, the model estimated an increase with 19–28% per week, with the highest rate in spring wheat (Table 4; Online Resource 3). For NIV and the sum of HT-2 and T-2 toxins, there were no significant effects of harvest week in any of the crops (Table 4; Online Resource 3).

**Table 4:** Effect of harvest date on *Fusarium* mycotoxin contamination with deoxynivalenol (DON), zearalenone (ZEN), nivalenol (NIV) and HT-2 and T-2 toxins of Swedish cereals. Analysis of deviance table

	**DON**						**ZEN**					**NIV**					**HT-2 + T-2**				
		*F* value	Df	Res. Df	*p* value		*F* value	Df	Res. Df	*p* value		*F* value	Df	Res. Df	*p* value		*F* value	Df	Res. Df	*p* value	
**Winter wheat**	Intercept	5.1	1	347	0.025	*	30.9	1	401	0.000	***	11.4	1	356	0.001	***	24.2	1	426	0.000	***
	Harvest week	23.1	1	435	0.000	***	42.5	1	430	0.000	***	0.9	1	383	0.341		0.5	1	449	0.466	
**Winter triticale**	Intercept	10.6	1	102	0.002	**	3.9	1	97	0.051		3.5	1	125	0.063		3.2	1	106	0.075	
	Harvest week	19.1	1	103	0.000	***	5.5	1	99	0.021	*	0.5	1	126	0.491		1.0	1	107	0.323	
**Spring wheat**	Intercept	8.8	1	134	0.004	**	10.5	1	148	0.002	**	4.8	1	46	0.033	*	18.2	1	138	0.000	***
	Harvest week	22.1	1	136	0.000	***	14.6	1	151	0.000	***	0.2	1	47	0.633		3.9	1	143	0.052	
**Spring oats**	Intercept	1.2	1	141	0.273		22.3	1	146	0.000	***	2.4	1	121	0.124		1.7	1	132	0.193	
	Harvest week	0.7	1	140	0.406		28.2	1	148	0.000	***	0.0	1	124	0.824		0.0	1	134	1.000	
**Springbarley**	Intercept	4.5	1	110	0.035	*	0.0	1	56	0.846		0.3	1	68	0.558		4.1	1	67	0.047	*
	Harvest week	0.2	1	112	0.648		0.1	1	59	0.797		0.4	1	71	0.545		0.5	1	70	0.479	

^*^p < 0.05; **p < 0.01; ***p < 0.001

### Correlation between mycotoxins and *Fusarium* species

**Fig. 5:**
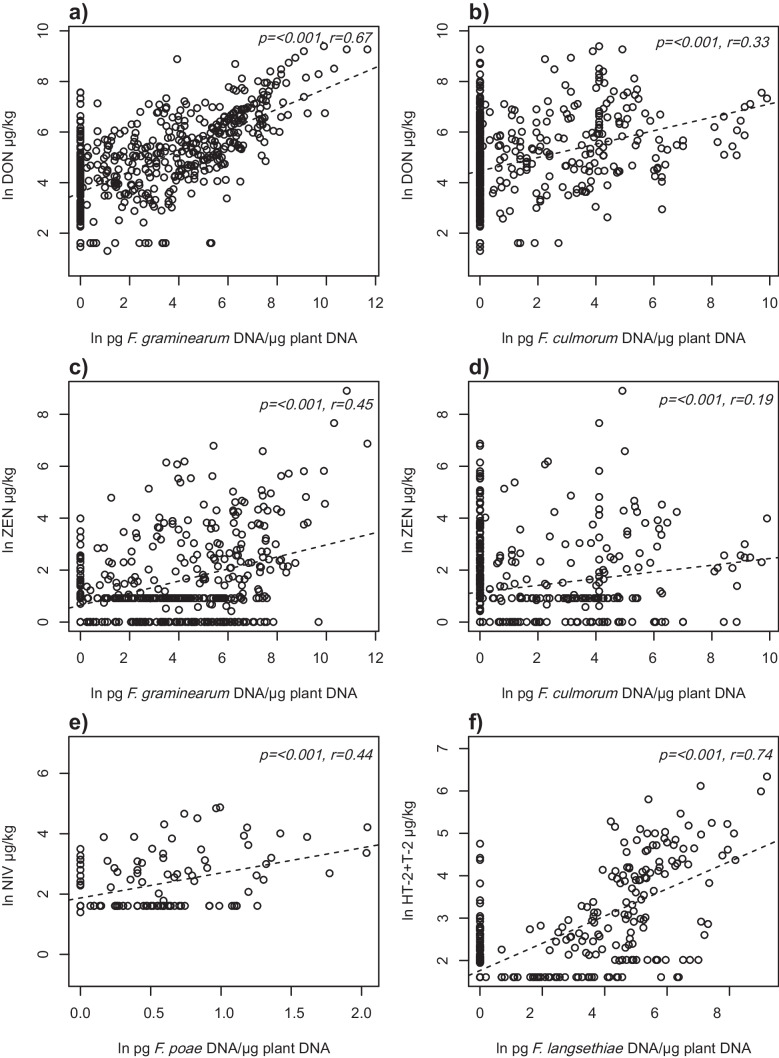
Correlations of mycotoxin content and different *Fusarium* species: **a** deoxynivalenol (DON) and *F. graminearum*, **b** DON and *F. culmorum*, **c** zearalenone (ZEN) and *F. graminearum*, **d** ZEN and *F. culmorum*, **e** nivalenol (NIV) and *F. poae* and **f** the sum of HT-2 and T-2 toxins and *F. langsethiae*. Both mycotoxin and *Fusarium* data are transformed with the natural logarithm (ln)

Regression analysis showed that there was a significant correlation between DON and *F. graminearum* (Fig. 5a). The same analysis was made for DON and *F. culmorum*, which also showed a significant correlation (Fig. 5b); however, the correlation was stronger for *F. graminearum*.

Regression analysis also revealed that ZEN correlated with *F. graminearum* (Fig. 5c). This was also true for ZEN and *F. culmorum* (Fig. 5d). Again, *F. graminearum* correlated better with ZEN than *F. culmorum*. The sum of *F. graminerum* and *F. culmorum* correlated only slightly better with DON (*p* ≤ 0.0001, *r* = 0.71) and ZEN (*p* < 0.001, *r* = 0.45) than *F. graminearum* alone.

For NIV, the correlation with the species *F. poae* was significant (Fig. 5e) but not with *F. graminearum* (*p* = 0.97, *r* = −0.002) or *F. culmorum* (*p* = 0.87, *r* = −0.007). For HT-2 and T-2 toxins, a significant correlation was found with *F. langsethiae* (Fig. 5f).

Relatively large variation was observed in all the correlation analyses between toxins and *Fusarium* species, where the amount of variation explained varied between 4 and 55%. Many samples contained low levels of fungal DNA and at the same time high toxin levels. The opposite was also common, high toxin levels and lower levels of fungal DNA in the same sample (Fig. 5).

**Fig. 6:**
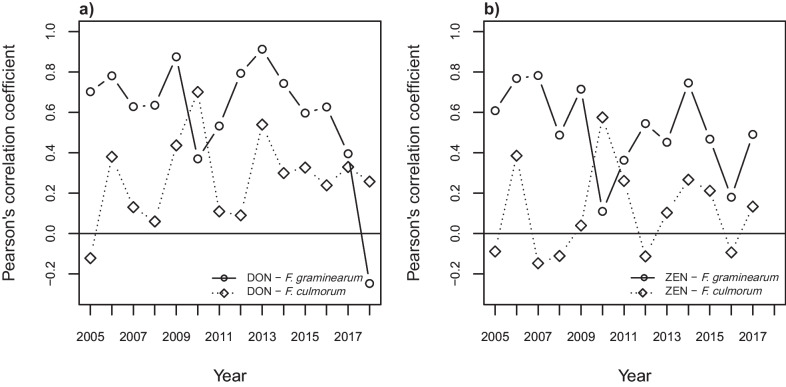
Yearly variation in correlations between mycotoxin and *Fusarium* DNA contamination for **a** deoxynivalenol (DON) and *F. graminearum*/*F. culmorum*, **b** zearalenone (ZEN) and *F. graminearum*/*F. culmorum*. No samples with ZEN over the limit of quantification were present in 2018

In order to better understand yearly variations in dominant species, Pearson’s correlation coefficient for *F. graminearum* and *F. culmorum* and DON and ZEN respectively was compared yearly (Fig. 6). *F. graminearum* dominated over *F. culmorum* fourteen out of 16 years. The exceptions were 2010, where *F. culmorum* was the most frequent species found in both DON and ZEN contaminated samples (see also Fig. 3b), and 2018, when only a positive correlation between DON and *F. culmorum* was observed.

## Discussion

A lower proportion of samples than expected exceeded legislative limits given that very severe problems with DON were reported from the cereal industry some years during the study period, especially 2011–2012 (Hartman and Börjesson 2012). Mycotoxin contamination levels in field trials may be lower than in practical agriculture due to several reasons. First, field trials are placed in homogenous parts of fields avoiding for instance depressions. It has been shown that *Fusarium* abundance is higher in more humid and cooler parts of a field (Schiro et al. 2018). Second, it can also be expected that field trials are harvested at a more optimal time, and that the smaller volumes of cereals from field trials are dried faster and stored for shorter time, limiting potential post-harvest toxin accumulation. Third, trials might be managed more intensively regarding for instance herbicide applications, fertilization and soil tillage. These factors may lead to an underestimation of mycotoxin contamination in field trials compared to farmers’ fields.

In Swedish commercial cereal production, DON has been considered the main *Fusarium* mycotoxin problem especially in the western part of the country corresponding to region ‘West’ in our analysis. The western area have had the most severe DON toxin problems in spring oats according to the cereal industry. In 2011, it was reported that half of the spring oats harvested for human consumption exceeded the EU limit for DON (Nilsson 2012). Analyses of mycotoxin data by the Swedish Food Agency from a 3-year survey 2009–2011 showed that not only spring wheat but also spring oats had the highest DON levels in samples collected in western Sweden (Lindblad et al. 2013; Fredlund et al. 2013). Our results identifying the highest DON levels in spring wheat in western Sweden are partly in contrast to these previous results and experiences from practice identifying the main problems in spring oats. One explanation may be that in practical agriculture, bread wheat may be more readily harvested than oats, while the difference in harvest date for the two crops in field trials may be less pronounced. Our data also included a longer time period than previous studies, and patterns of dominant species and toxins change over time.

Both DON and ZEN occurred in the highest concentrations in the region ‘West’. Region ‘West’ receives the largest amounts of precipitation of the five regions, which may be one explanation of the higher toxin levels in this area. A previous study in winter wheat showed that *F. graminearum* was present in higher abundance in the western part of the country, compared to the northeastern part corresponding to region ‘North’ in our study (Karlsson et al. 2017).

In contrast to DON and ZEN, HT-2 and T-2 toxins occurred to a greater extent in region ‘North’ compared to the other regions, and we speculate that the general less humid conditions in this area may be an explanation. It has been suggested that *F. langsethiae* is disfavoured by not only wet weather conditions but also cooler temperatures (Imathiu et al. 2013). Another characteristic of region ‘North’ is that it has the highest soil clay content of the five regions. A negative correlation between clay content and *F. graminearum* was identified in a study in Norway, but no relationship between *F. langsethiae* and soil texture was found (Bernhoft et al. 2012), but weather conditions are probably more important explaining regional variation (Landschoot et al. 2012).

Edwards (2017) shows in a UK survey of oat grains that the sum of HT-2 and T-2 toxins dominates clearly over DON levels. This is in contrast to Sweden where DON is the main problem. Interestingly, spring oats had a significantly lower level of HT-2 and T-2 toxins than winter oats (Edwards 2017). As Sweden grow spring oats, this may be the reason for low HT-2 and T-2 levels in the present study. The two types of flower at different time and the earlier flowering period for the winter type may be correlated with the biology of the causing fungus *F. langsethiae.* In a survey in Norway, HT-2 and T-2 toxins were more frequently detected in oats compared to spring wheat. This was in contrast to DON which was detected in similar frequency in both crops (Hofgaard et al. 2016). Norway is also producing spring oats so the more frequent presence of HT-2 and T-2 toxins in Norwegian oats compared to Swedish oats is more likely due to other factors, such as environmental conditions or *Fusarium* susceptibility of genotypes grown.

In the present study, spring crops had in general higher toxin contamination levels than winter crops. This characteristic was especially evident for DON where spring wheat had significantly higher levels than winter wheat. In a Finish survey, the highest median DON concentrations were detected in oats followed by spring wheat and barley, while winter wheat and rye had lower levels (Hietaniemi et al. 2016). Also HT-2 and T-2 toxins and NIV followed similar patterns where the spring crop oats and barley had the highest levels in our study. A study in Lithuania found that spring wheat and barley were more often contaminated with several mycotoxins compared to winter wheat, rye and triticale (Mankevičienė et al. 2011). These results may be explained by differences in the causing *Fusarium* species’ biology and ecological preferences, or to what extent the different crops are susceptible, or a combination of the two, for instance, the growth and reproduction of the fungus over the season compared to the time of flowering where the crops are most susceptible. But it is difficult to separate genetic factors and agronomic factors (Opoku et al. 2018).

Official field trials are generally readily harvested after the crop has reached maturity. However, during the busy harvest period, there needs to be a prioritization of individual trials, and weather conditions may be unfavourable, leading to a later harvest than optimal for some trials. Delayed harvest was associated with significantly higher levels of DON and ZEN in spring and winter wheat, winter triticale and for ZEN also in spring oats. We analysed the effect of calendar week on mycotoxin contamination. However, the data did not allow evaluation of the grade of maturity of different varieties at harvest. Calendar week may therefore be a coarse measure of delayed harvest and the results should be interpreted cautiously. However, our results are in line with previous findings. Edwards and Jennings (2018) identified delayed harvest compared to the long-term average harvest date as a risk factor for DON and ZEN contamination of winter wheat grown in the UK. The length between flowering and harvest was associated with increased DON levels in wheat in a study using data from Sweden, Finland, Norway and the Netherlands (van der Fels-Klerx et al. 2012).

In a recent study in the Baltic countries, precipitation at ripening was identified as a risk factor for DON contamination in several crops (Marzec-Schmidt et al. 2021). This indicates that delayed harvest may be especially problematic under rainy conditions, or that rain is often causing delayed harvest, and the effect of these two factors may be difficult to separate. Post-anthesis rainfall and delayed harvest have been shown to increase ZEN levels in winter wheat and that these two factors interacted positively (Kharbikar et al. 2015). The same study showed no effect of late harvest on DON and that rainfall had inconsistent effects. This may be explained by leaching of DON caused by high levels of rainfall, while ZEN is not as motile (Edwards et al. 2018).

Our data did not show any influence of delayed harvest on the toxins NIV, HT-2 and T-2. Therefore, delayed harvest may not be a risk factor for contamination of these toxins, but this result should be confirmed by other studies.

During the present analysed 16-year period, *F. graminearum* dominated over *F. culmorum* as a DON producer. More than 20 years ago, *F. culmorum* was considered to be the main DON producer in Northern Europe but today, *F. graminearum* is the most important species (Waalwijk et al. 2003; Nielsen et al. 2011; Bilska et al. 2018; Hofer et al. 2019). Our result that *F. graminearum* is the dominating DON producer is in accordance with the results from a 3-year survey made by the Swedish Food Agency (Lindblad et al. 2013; Fredlund et al. 2013). In our material, *F. culmorum* was the dominating species in 2010, and had a better correlation to DON content this year, while there was a complete shift to *F. graminearum* in 2011. This was most apparent in the western part of the country (Fig. 3b). Interestingly, 2011 was also the year when severe problems with DON were reported by the industry, and we conclude that *F. graminearum* was contributing the most to the high DON levels this year. However, from 2013, *F. culmorum* increased in importance again. In 2018, a year with a very hot summer and severe drought in Sweden, there was a better correlation between DON and *F. culmorum* than for *F. graminearum*. These results indicate that the relationship between the two species is not static and that the most important species may change.

Our results indicate that *F*. *poae* rather than *F. culmorum* or *F. graminearum* was the main producer of NIV during the study period. This was also reported from earlier studies in Sweden (Pettersson et al. 1995; Lindblad et al. 2013; Fredlund et al. 2013) and in other Nordic countries (Langseth and Rundberget 1999; Hietaniemi et al. 2016). Interestingly, in contrast to the other toxins, there were no regional differences in NIV contamination. The NIV-producing chemotypes of *F. culmorum* and *F. graminearum* have been identified in Western Europe, including Denmark but not in Norway or Finland (Pasquali et al. 2016). The chemotype distribution has to our knowledge not been investigated in Sweden.

Several studies have recently reported an increased frequency of *F. poae* both in Europe (Hietaniemi et al. 2016; Beccari et al. 2017) and Northern America (Valverde-Bogantes et al. 2019) and it is speculated that *F. poae* is favoured by drier conditions compared to *F. graminearum*. If so, an increase of NIV may be expected due to climate change.

The best correlation between a toxin and *Fusarium* species in our study was between *F. langsethiae* and the sum of HT-2 and T-2 toxins. This toxin may also be produced by *F. sporotrichioides* which was not surveyed, but has been detected in Sweden earlier but at lower levels than *F. langsethiae* (Fredlund et al. 2013).

The correlation between fungal DNA and mycotoxin content was sometimes weak in our study. Although a species or isolate may have the genes to produce a certain mycotoxin, toxin production is dependent on environmental factors such as temperature and humidity and competition with other *Fusarium* species present (Xu et al. 2007; Gautam and Dill-Macky 2012; Nazari et al. 2014). Environmental conditions triggering or inhibiting mycotoxin production may explain the presence of samples with low content of fungal DNA and high content of mycotoxin, and vice versa, in our dataset.

There are several methods for handling values below the limit of detection or limit of quantification (LOD/LOQ). It is common to use different forms of substitution, here we replaced the values < LOQ with LOQ/2. It is important to note that the choice of substitution method may significantly influence metrics such as mean values, if a large proportion of data fall below the LOD/LOQ (EFSA 2010; WHO 2020). This is important when calculating for instance dietary exposure levels, but we estimate that this type of bias is less relevant for the aim of our study, which is to compare the effects of different factors on mycotoxin contamination.

Our study showed that different *Fusarium* toxins have different distributions depending on both crop and geographical location. DON and ZEN had a similar distribution with highest levels in spring wheat in the most humid western part of Sweden. NIV, HT-2 and T-2 toxins occurred to a greater extent in spring barley and spring oats than in the other cereals. HT-2 and T-2 toxins were more frequent in the northernmost part of the sampling area. Later harvest was significantly associated with elevated levels of DON and ZEN in several crops. This knowledge can be used by farmers to prioritize crops with the highest risk of mycotoxin contamination in their region at harvest, reducing the risk of mycotoxins accumulating and entering the food chain. The importance of different *Fusarium* species and toxins varied over time, which highlights the need for continuous monitoring to detect and respond to new patterns of mycotoxin contamination.

## Supplementary Information

Below is the link to the electronic supplementary material.

Supplementary file1 (DOCX 310 KB)
