# Supplementary material for: Temporal and spatial dynamics of Fusarium spp. and mycotoxins in Swedish cereals during 16 years
Source: Mycotoxin Res. 2022 Oct 24;39(1):3–18. doi: 10.1007/s12550-022-00469-9 (PMC10156870; doi:10.1007/s12550-022-00469-9)
Supplement: Supplementary file 1 — Supplementary file1 (DOCX 310 KB) [file 12550_2022_469_MOESM1_ESM.docx]

Online resources

**Online resource 1** Multiple reaction monitoring (MRM) transitions and compound-dependent parameters for quantification during LC–MS/MS analysis

| **Compound** | **Q1 (m/z)** | **Q3 (m/z)** | **DP (V)** | **EP (V)** | **CE (V)** | **CXP (V)** | **Names** |
| --- | --- | --- | --- | --- | --- | --- | --- |
| HT-2 | 447 | 285 | 50 | 5 | 30 | 4 | HT-2 |
| HT-2 IS (^13^C_22_) | 469 | 300 | 50 | 5 | 30 | 4 | HT-2 |
| T2 | 489 | 245 | 50 | 7 | 30 | 4 | T-2 |
| T2 IS (^13^C_22_) | 513 | 260 | 50 | 7 | 30 | 4 | T-2 |
| DON | 295 | 265 | -30 | -11 | -15 | -3 | deoxynivalenol |
| DON IS (^13^C_22_) | 310 | 279 | -30 | -11 | -15 | -3 | deoxynivalenol |
| NIV | 311 | 281 | -40 | -10 | -15 | -2 | nivalenol |
| NIV IS (^13^C_22_) | 326 | 295 | -40 | -10 | -15 | -2 | nivalenol |
| ZEA | 317 | 131 | -60 | -11 | -40 | -10 | zearalenone |
| ZEA IS (^13^C_22_) | 335 | 139 | -60 | -11 | -40 | -10 | zearalenone |

Abbreviations: Q1 and Q3 = m/z ratios from quadrupole 1 and quadrupole 3; CE, collision energy; CEP, cell entrance potential; CXP, cell exit potential; DP, declustering potential; EP, entrance potential.


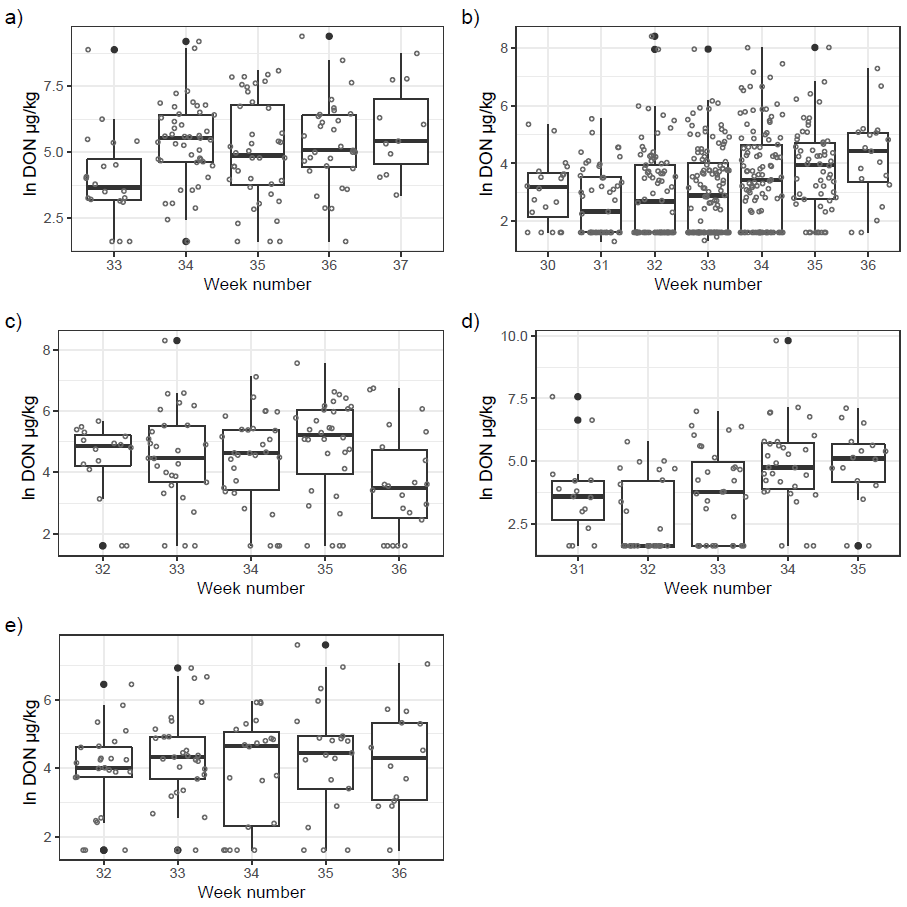


**Online resource 2** DON contamination depending on harvest week in a) spring wheat, b) winter wheat, c) spring oats, d) winter triticale and e) spring barley. Only weeks with 10 or more samples are shown. Mycotoxin data are transformed with the natural logarithm (ln)


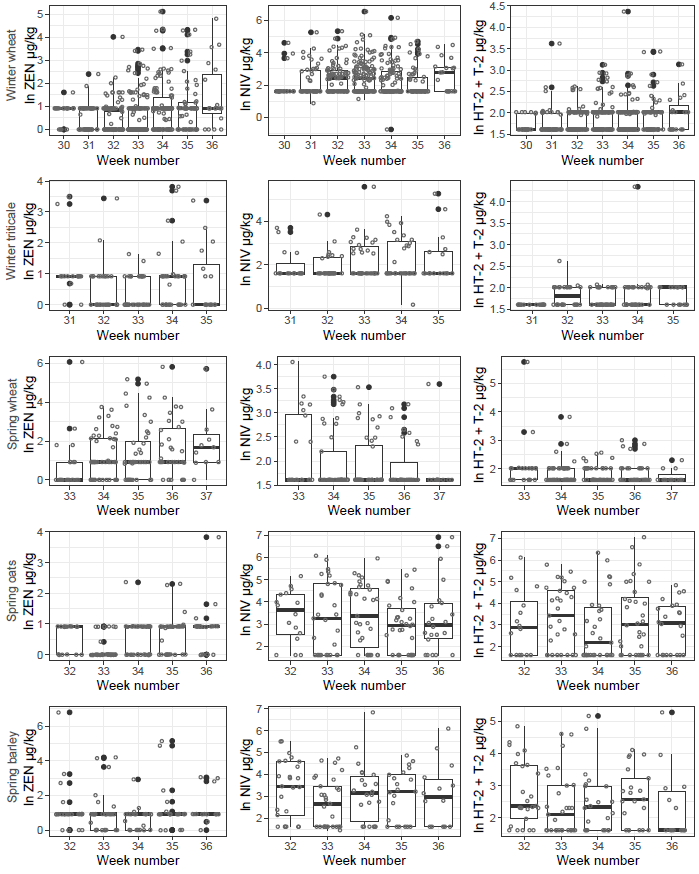


**Online resource 3** Mycotoxin contamination depending on harvest week in different crops. Only weeks with 10 or more samples are shown. Mycotoxin data are transformed with the natural logarithm (ln). DON = deoxynivalenol, ZEN = zearalenone, NIV = nivalenol
